# Convolutional Neural Networks and Heuristic Methods for Crowd Counting: A Systematic Review

**DOI:** 10.3390/s22145286

**Published:** 2022-07-15

**Authors:** Khouloud Ben Ali Hassen, José J. M. Machado, João Manuel R. S. Tavares

**Affiliations:** 1 Faculdade de Engenharia, Universidade do Porto, Rua Dr. Roberto Frias, s/n, 4200-465 Porto, Portugal; up202100780@edu.fe.up.pt; 2 Departamento de Engenharia Mecânica, Faculdade de Engenharia, Universidade do Porto, Rua Dr. Roberto Frias, s/n, 4200-465 Porto, Portugal; jjmm@fe.up.pt

**Keywords:** computer vision, deep learning, people counting, sparse datasets, crowded datasets

## Abstract

The crowd counting task has become a pillar for crowd control as it provides information concerning the number of people in a scene. It is helpful in many scenarios such as video surveillance, public safety, and future event planning. To solve such tasks, researchers have proposed different solutions. In the beginning, researchers went with more traditional solutions, while recently the focus is on deep learning methods and, more specifically, on Convolutional Neural Networks (CNNs), because of their efficiency. This review explores these methods by focusing on their key differences, advantages, and disadvantages. We have systematically analyzed algorithms and works based on the different models suggested and the problems they are trying to solve. The main focus is on the shift made in the history of crowd counting methods, moving from the heuristic models to CNN models by identifying each category and discussing its different methods and architectures. After a deep study of the literature on crowd counting, the survey partitions current datasets into sparse and crowded ones. It discusses the reviewed methods by comparing their results on the different datasets. The findings suggest that the heuristic models could be even more effective than the CNN models in sparse scenarios.

## 1. Background

Because of the fast growth of the world’s population, and situations where crowds occur, such as concerts, political speeches, rallies, marathons, and stadiums, crowd counting is becoming an active research topic in computer vision [1]. The task of crowd counting, defined as determining the number of people in a crowd, would help in many fields, such as in video surveillance for safety reasons, human behavior analysis, and urban planning [2,3,4,5]. Many approaches have been proposed in the literature to solve this problem, which generally can be split into four categories: detection, regression, density estimation, and approaches based on convolutional neural networks (CNNs). This article highlights the main architectures and models of crowd counting to explain the evolution of this problem and the solutions proposed in the literature.

## 2. Introduction

As mentioned previously, this review divides the crowd counting models into four categories. Starting with the detection-based method, the principle behind it to use a moving window as a detector to identify and count how many persons are in an input image [6]. Although these methods work well for detecting faces, they do not perform sufficiently well on crowded images as most target objects are not clearly visible. Counting by detection is categorized into five types: monolithic detection [7,8,9], part-based detection [10,11], shape matching [12,13], multi-sensor detection [14], and transfer learning [15,16].

Since counting by detection is not very precise when factors such as dense crowds and high background clutter appear, researchers proposed a regression method [17] to overcome these problems, where neither segmentation nor tracking individuals are involved. First, it extracts the low-level features such as edge details and foreground pixels and then applies regression modelling to them by mapping the features and the count.

Clustering models are about selecting and gathering feature points or trajectories of feature points. These methods use unsupervised learning to identify each moving entity by an independent motion [18].

Among existing approaches, CNN based methods [19,20] have proved their efficiency and exhibit the best results for the crowd counting task. The general concept behind using deep convolutional networks is to scan the input image to understand its different features and then to combine the different scanned local features to classify it. According to the used network architecture, crowd counting models can be classified into: basic CNN [21,22], multi-column [23,24,25], and single column-based methods [26,27,28,29,30].

This review article presents a thorough study of the aforementioned different approaches by understanding their concepts and architectures, highlighting the best scenarios to use, and their advantages and disadvantages. The reviewed solutions are mainly divided into heuristic and deep learning methods. The reason behind this partitioning is that, after going through solutions in the literature, the most observed characteristic is that approaches are progressing in architecture, and the most highlighted shift is moving to deep learning methods.

Different scenarios exist in the literature for datasets, such as sparse and crowded. This article also proposes a benchmark study by comparing the results of the reviewed methods on these datasets.

**Figure 1:**
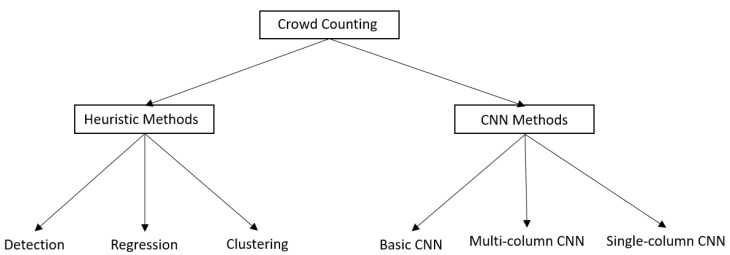
Overall structure of the current review study.

This article is organized according to the following structure: Section 2 explains the methodology used to search for the most relevant articles to be reviewed. Section 3 presents the existing and most used traditional methods for crowd counting, namely, the heuristic methods. Section 4 approaches the deep learning methods; it includes an explanation based on the architectures of the different approaches. In Section 5, a further study into the different types of datasets used for crowd counting is presented. Thereafter, the results and discussion section describes the models found for crowd counting task and their efficiency in different scenarios. Finally, the Future Scope and Challenges section explores the future work and the challenges faced for the crowd counting task. Figure 1 summarizes the taxonomy followed in this review to better understand its organization.

## 3. Literature Search Method

This section describes the process of including and excluding research articles in the current review in order to have higher transparency of the used methodology and the selection criteria. The main goal was to sort out the critical works on crowd counting based on different approaches and architectures. For that, the following aspects were considered:

- For which situation was the task of crowd counting addressed?
- Which datasets were used? What was the nature of the elements in the dataset? Were they persons, animals, or other objects?
- Which architecture was used?
- What metrics were used for evaluation?

### 3.1. Inclusion Criteria

The studies included in the current review were defined based on the following criteria: they should tackle the crowd counting problem by explaining the architecture used in the solution, the reasons behind using it, its novelty, and its limitations. The studies should include the context where they were trying to solve the crowd counting problem. At least one of the datasets used for experimentation should consist of images that contain humans. The research studies should include quantitative or qualitative results to measure their effectiveness, and the studies should be in English.

### 3.2. Databases and Search Steps

A systematic literature search was executed in the ScienceDirect and Scopus databases with the following keywords in multiple combinations: “crowd counting”, “crowd estimation”, “crowd detection”, “people counting”, and “computer vision for crowd counting”.

**Figure 2:**
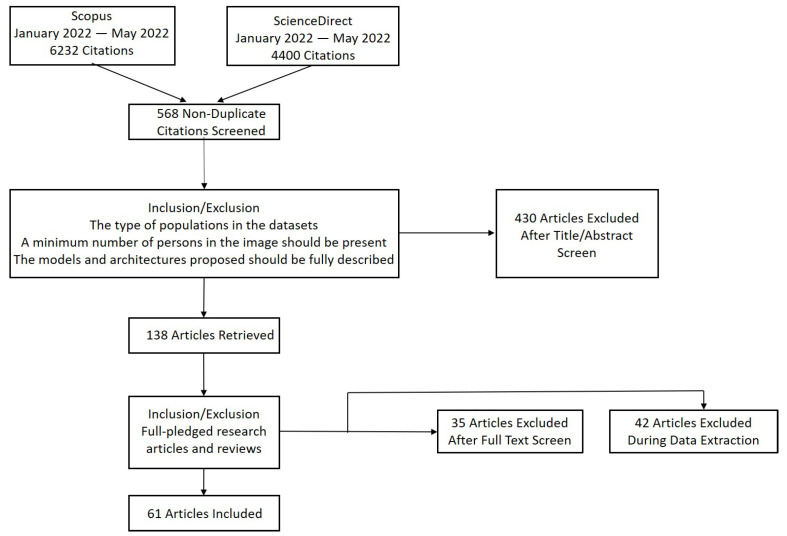
PRISMA diagram showing the results of the executed literature search.

Consequently, 568 documents were obtained at the time of the search between reviews and research articles, based on title and abstract analysis, most of which were completely unrelated to the subject. One hundred thirty-eight articles were then selected for further analysis based on the following inclusion criteria: the type of population in the datasets, i.e., humans, should be included in the images, and not only animals or other objects. A minimum number of persons in the image should be present to discuss counting. Otherwise, it would be a problem of visualization or maybe behavior detection. The models and architectures proposed should be fully described in order to explain their limitations and advantages. It was also necessary to consider clear guidelines for using metrics for evaluation and comparing related models on different datasets. Finally, using an excel sheet, the selected articles were organized and filtered to understand their contribution, ranking their information in terms of the article’s year, type, authors, institute, proposed model, used datasets, and used performance metrics. Figure 2 illustrates the adopted literature searching process with the results obtained after each step.

## 4. Heuristic Models

Early methods of this category estimate the pedestrian number via heuristic methods [31], for instance detection-based, regression-based, and density-estimation-based methods. This section explains in more detail these models and how they work.

### 4.1. Detection Based Methods

Earlier works on crowd counting were focused on detection-based methods to determine the number of people in the crowd [32,33,34]. They mainly detect each target person in a given image using specific detectors. In the following paragraphs, an explanation of these methods with some examples is given.

Monolithic detection: it is considered a typical pedestrian detection approach that trains the classifier, utilizing the entire body of a set of pedestrian training images [7,8,9,31]. In order to represent the entire body’s appearance, common features are used: Haar wavelets, gradient-based features, edgelet, and shapelets. As to the classification, several classifiers were used:

- Non-Linear: Similarly to RBF, Support Vector Machines (SVMs) present good quality while suffering from low detection speed.
- Linear: more commonly used classifiers such as boosting, linear SVMs, or Random Forests [35].

A trained classifier is applied in a sliding window fashion across the image space to catch pedestrian candidates. A monolithic detector can generate good detection in sparse scenes. However, it suffers in congested locations where it is impossible to avoid occlusion and scene clutter.

Part based detection: consists in constructing boosted classifiers for precise body parts, for instance the head and the shoulder, to count the people in the monitored region [10,11,36]. The idea is to include the shoulder region with the head to account for the real-world scenario better. Another method relies on a head detector to count people [37], which is based on finding interest points using gradient information from the greyscale image located at the top of the head region in order to reduce the search space.

Compared to monolithic detection, part-based detection relaxes the stringent hypothesis regarding the visibility of the whole body. As a result, it is more robust in crowds but it always suffers from the occlusion problem.

Shape matching: the idea is to detect the body shapes of the peoples in the crowd to count them. Zhao et al. [12] presented a set of parameterized body shapes formed of ellipses and zeros to estimate the number and shape configuration that best presents a given foreground mask in a scene, employing a stochastic process. Ge and Collins [13] developed the idea by permitting more flexible and realistic shape prototypes than only the simple geometric forms presented in [12]. The learned shape prototypes are more accurate than simple geometric shapes. The method proposed by Ge and Collins [13] can detect varying numbers of pedestrians under different crowd densities with reasonable occlusion.

Multi-sensor detection: When numerous cameras are available, one can also include multi-view information to handle visual ambiguities generated by inter-object occlusion. For instance, ref. [14] worked on extracting the foreground human silhouettes from the images under analysis in order to set bounds on the number and potential areas where people exist. The issue with these methods is that a multi-camera configuration with overlapping views is not always available in many possible applications.

Transfer learning: it is about transferring the generic pedestrian detectors to a new scene without human supervision. This solution faces the problems of the variations of viewpoints, resolutions, illuminations, and backgrounds in the new environment. A key to overcome these challenges is proposed in [15,16], by using multiple parameters such as scene structures, spatial-temporal occurrences, and object sizes to determine positive and negative examples from the target scene in order to iteratively adjust a generic detector.

### 4.2. Regression Methods

Because of the difficulty of detection-based models in dealing with highly dense crowds and high background clutter, researchers introduced regression-based approaches, which are inspired by the capacity of humans to determine the density at first sight without the need to enumerate how many pedestrians are in the scene under analysis [17]. Such a method counts people in crowded scenes by discovering a direct mapping from low-level imagery features to crowd density. First, it extracts global features [38]: texture [39], gradient or edge, or local features [40], such as Scale-invariant Feature Transform (SIFT), Local Binary Patterns (LBP), Histogram of Oriented Gradients (HOG), and Gray Level Co-occurrence Matrix (GLCM). After the feature extraction step, it trains a regression model to indicate the count given the normalized features. Among the regression techniques, one can mention: linear regression [41], piecewise linear regression [17], and Gaussian mixture regression [42].

Another approach from Idrees et al. [43] considered that, in highly crowded scenes, there is no feature or detection approach reliable enough to deliver sufficient information for a precise counting because of the low resolution, severe occlusion, foreshortening, and perspective problems. Furthermore, the presence of a spatial relationship is used in constraining the count estimates in neighboring local regions, and it is suggested that the extraction of features be performed using different methods to catch the different information. Table 1 summarizes some of the regression-based methods.

### 4.3. Clustering Based Methods

Another alternative technique is counting by clustering. The idea is to decompose the crowd into individual entities. Each entity has unique patterns that can be clustered to determine the number of individuals [31].

Rabaud et al. [48], used a simple yet effective tracker, the Kanade–Lucas–Tomasi (KLT), to extract a large set of low-level features in pedestrian videos. It is proposed as a conditioning technique for feature trajectories to identify the number of objects in a scene. A complementary trajectory set clustering method was also introduced. The method can only be applied to crowd-counting videos. Three different real-world datasets were used to validate and determine the method’s robustness: USC, Library, and Cells datasets [49].

Brostow et al. [50], proposed a simple unsupervised Bayesian clustering framework to capture people in moving gatherings, the principal idea being to track local features and group them into clusters. The algorithm tracks simple image features and groups them into clusters defining independently-moving entities in a probabilistic way. The method uses space-time proximity and trajectory coherence via image space as the only probabilistic criteria for clustering. This solution came instead of determining the number of clusters and setting constituent features with supervised learning or a subject-specific model. The results were encouraging from crowded videos of bees, ants, penguins, and most humans.

Rao et al. [51], explained the importance of crowd density estimation in a video scene to understand crowd behavior by implementing a crowd density estimation method based on clustering motion cues and hierarchical clustering. For motion estimation, the approach integrates optical flow. It employs contour analysis to detect crowd silhouettes and clustering to calculate crowd density. It starts by applying a lens correction profile to each image frame, followed by pre-processing the frames to remove noise. A Gaussian filter is applied to suppress high amplitude edges. Finally, the foreground pixels are mapped to crowd density by clustering the motion cues hierarchically. For evaluation, three datasets were used: MCG, PETS, and UCSD.

Antonini et al. [52], worked on video sequences to improve the automatic counting of pedestrians. A generative probabilistic approach was applied to better represent the data. The main goal was to analyze the computed trajectories, find a better representation in the Independent Component Analysis (ICA) transformed domain, and apply clustering techniques to improve the estimation of the actual count of pedestrians in the scene. The advantage of using the ICA generative statistical model is in reducing the influence of outliers.

## 5. Deep Learning Methods

Because of the CNN architecture’s efficiency in many tasks, including crowd counting, recent researchers used CNN as the base framework of their work. The general concept is to understand the various features of the image under analysis by browsing its content from left to right or top to bottom, and then combining the different scanned local features in order to classify it. A CNN includes three layers: convolutional layer, pooling layer, and fully connected layer [53,54,55].

- Convolutional layer: the primary role of this layer is to apply filters to detect features in the input image and build numerous feature maps to help identify or classify it. After every convolution operation, a linear function, the ReLU activation, is applied to replace the negative pixel values with zero values in the feature map.
- Pooling layer: this step takes the output feature map generated by the convolution. The goal is to reduce the complexity for further layers by applying a specific function such as the max pooling.
- Fully connected layer: every neuron from the previous layer is connected to every neuron on the next layer to generate the final classification result.

**Figure 3:**
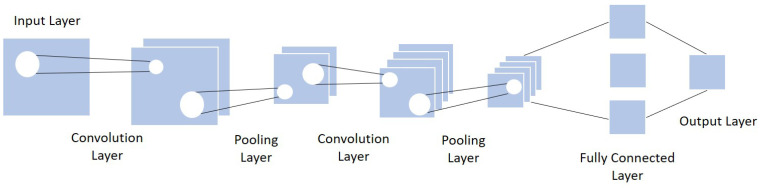
Usual CNN architecture (adapted from [56]).

Figure 3 shows the basic architecture of a CNN.

Table 2 details each usual CNN layer with its actions, parameters, inputs and outputs.

According to the architecture of the used CNN, crowd counting methods can be divided into basic CNN, multi column, and single column networks.

### 5.1. Basic CNN

**Figure 4:**
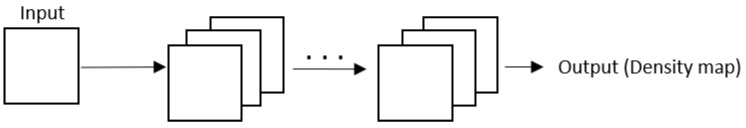
General structure of the Basic CNN architecture.

Among the CNN architectures, one has the basic CNN with its light network. It adopts the primary CNN layers: the convolutional layer, the pooling layer, and the fully connected layer. Figure 4 presents a simplified structure of the fundamental CNN.

Wang et al. [21] proposed a solution that can provide good results in high-density crowds, unlike the traditional methods that would fail in these scenarios, consisting of a deep regression network in crowded scenes using deep convolutional networks. The basic CNN architecture allows for efficient feature extraction. Since other objects can exist in dense crowd images, such as buildings and trees, influencing performance, the goal was to feed the CNN with negative samples to reduce false alarms. Few collected images without people were considered, and their regression score was set as 0 (zero), making the method more robust. The UCFCC dataset was used to evaluate the approach’s efficacy. A comparison between the CNN network with and without negative samples was performed. The method achieves almost 50% improvement.

Fu et al. [22] improved the speed and precision of the original approach by firstly removing some redundant network connections in the feature maps and, secondly, designing a cascade of two ConvNet classifiers:

- Optimizing the connections: the multi-stage ConvNet increases the number of features in the final classifier, and the connections seriously increase the calculation time during the training and detection phases. Some redundant connections among two similar feature maps were observed, so these extra connections were removed based on a similarity matrix to accelerate the speed.
- Cascade classifier: samples with complicated backgrounds are always hard to classify. The idea is to pick out those complex samples and train them individually and, after that, send them to a second ConvNet classifier to obtain the final classification result.

The three datasets used to evaluate this method were the PETS 2009, Subway, and Chunxi Road datasets, and the experiments confirm its excellent performance.

### 5.2. Multi column CNN

**Figure 5:**
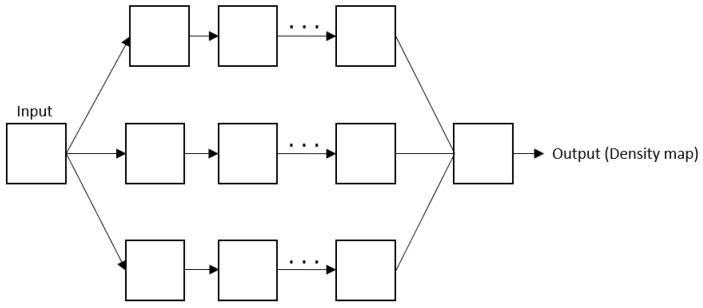
Overall architecture of the multi-column CNN.

To solve the variation problem, researchers have resorted to a multi-column architecture. Despite being harder to train, it proved its efficiency in specific situations. It consists of using more than one column to catch multi-scale information. Figure 5 represents the overall architecture of the multi-column CNN.

MCNN: Development of a multi-column CNN method to count the crowd in a single image from any perspective [23]. The application of an MCNN architecture with three columns occurs since each one corresponds to a filter with different sizes of receptive fields: large, medium and small, so that the features could adapt to significant variations in people. Moreover, to avoid distortion, a convolution layer with a filter size of 1 × 1 replaces a fully connected layer. It is flexible to inputs of different sizes. To test this method, a new large-scale dataset named Shanghaitech was introduced, containing two parts: part A and part B. In addition to Shanghaitech, the UCF CC 50, WorldExpo’10, and UCSD datasets were used to evaluate the proposed method. Compared to the existing methods at that time for crowd counting, their solution outperforms all the results.

CrowdNet: to forecast the density map for a provided crowd image, this method combines deep and shallow fully convolutional networks [24]. The shallow is to capture the low-level features with a large-scale variation: head blob patterns appearing from individuals far from the camera, and the deep one captures the high-level semantic details: faces/body detectors.

Because most datasets used for crowd counting have restricted training samples while deep learning-based approaches need extensive training data, the researchers opt for data augmentation by sampling patches from the multi-scale image representation to make the built models more potent to crowd variations. Therefore, the CNN is guided to learn scale-invariant representations. One of the most challenging datasets was used, the UCF CC 50, allowing the CNN to obtain competitive evaluation results.

RANet: starts from the problem that density estimation methods for crowd counting serve pixel-wise regression without accounting for the interdependence of pixels explicitly, which leads to noisy and inconsistent independent pixel-wise predictions [25]. To solve this issue, it was suggested to capture the interdependence of pixels thanks to a Relational Attention Network (RANet) with a self-attention mechanism by accounting for short-range and long-range interdependence of pixels. These implementations are Local Self-attention (LSA) and Global Self-attention (GSA).

In addition, features from LSA and GSA have different information for each part. The researchers introduced a relation module to link those features and reach better instructive aggregated feature representations using intra-relation and inter-relation. The datasets used to evaluate their model were the ShanghaiTech A and B, UCF-CC-50, and UCF-QNRF datasets.

### 5.3. Single Column CNN

**Figure 6:**
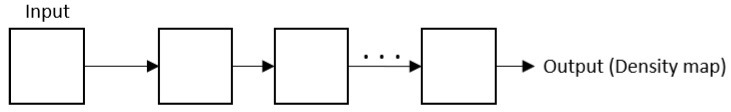
General structure of the single-column CNN.

This architecture consists of using only one single and deeper column to decrease the network’s complexity. Figure 6 depicts the single-column CNN.

CSRNet: to have a better understanding of the highly congested scenes, a pure, fully convolutional network (CSRNet) was proposed [27]. The architecture of this method consists of 2D feature extraction by resorting to a CNN as the front-end layer. Moreover, a dilated convolution layer is the back-end used to extract more profound features without losing resolution and enlarge the receptive fields. The front-end CNN is identical to the first ten layers of VGG-16 with three pooling layers. The choice of VGG-16 was because of its powerful transfer-learning capacity and flexible architecture.

The back-end CNN is a sequence of dilated convolutional layers, where the last layer with a 1 × 1 dimension produces a density map. Dilated convolution uses sparse kernels to alternate the pooling and convolutional layers. Due to these characteristics, the receptive field is larger without augmenting the number of parameters or the computational demand.

D-ConvNet: the abbreviation of De-correlated ConvNet. It enhances the generalization capacity of the ensemble models by taking the benefit of negative correlation learning (NCL) with a pack of weak regressors with convolutional feature maps [29].

SaCNN: refers to the abbreviation of a scale-adaptive CNN. Its contribution is building a single-backbone network with a single filter size [26]. It combines feature maps of multiple layers to solve the problem of changes in pedestrian scale and perspective. Faster training is obtained due to fewer parameters and the requirement of fewer training data by using multi-scale layers that share the same low-level parameters and feature representations. Finally, two loss functions aiming to optimize the method consist firstly of the density map loss, and the second is relative count loss, which helps reduce the variance of the prediction errors and improves the network generalization in the presence of sparse crowd scenarios. In addition to using the ShanghaiTech and UCF CC 50 datasets, a new dataset was used: the SmartCity dataset, which contains 50 images collected from ten cities, with both outdoor and indoor scenes.

TedNet: it is an encoder-decoder network architecture. The model integrates multiple decoding paths to catch multi-scale features and obtain the supervised information by exploiting dense skip connections [28]. In addition, it introduced a combinational loss comprising local coherence and spatial correlation loss to reduce the gradient vanishing problem and improve the back-propagation ability.

CNN with pixel-wise attention mechanism: the method is composed of three modules. The first adopts a foreground extraction approach to stop the noise and outliers generated by the background. The second module uses a pixel-wise attention technique to solve the non-uniform distribution of people. Finally, a unique single-column network, which was designed with much fewer parameters and can achieve similar results, is used [30]. It helps to reduce computing complexity. In addition, a new large-scale crowd-counting image dataset obtained from surveillance cameras, the WJ dataset, was proposed, which contains different weather atmospheres, illumination conditions, scales, and image conditions.

Counting people in a crowd is a complex process; over the years, researchers tried to improve the proposed methods [57]. The development of new methods can be driven by the advantages and disadvantages of previous ones. Table 3 summarizes different models’ weak and strong characteristics. The presented comparison is based on the used architectures instead of the methods’ specifics, because solutions using the same architecture have the same main advantages and disadvantages.

## 6. Datasets

Methods for crowd counting and density estimation were assessed on various datasets containing different objects, such as humans and pets, cars, and only humans. The datasets selected for review in this article contain only humans or a mix of humans and other kinds of objects. Additionally, the number of objects of interest in the crowd varies among the datasets, it therefore being usual to divide them into sparse and crowded datasets, which are detailed in the following.

### 6.1. Sparse Datasets

This subsection introduces the sparse datasets: UCSD, Mall, Shangaitech Part B, and SmartCity.

UCSD: it is a pedestrian dataset containing a 2000 frame movie acquired by a stationary digital camcorder on the UCSD campus viewing a pedestrian walkway [17,58]. There are 49,885 pedestrian occurrences, each image has a resolution of 238 × 158 pixels, and the crowd count ranges from 11 to 46 persons per image.

Mall: this dataset includes 2000 frames of footage at a resolution of 320 × 240 pixels acquired by a shopping center surveillance camera [38]. The total number of pedestrian instances is 62,325, varying from 13 to 53 in each image frame.

Shangaitech part B: it is part of the ShanghaiTech dataset that includes 1198 labelled images from 330,165 individuals [23]. ShanghaiTech part B contains 716 images acquired in the busy streets of Shanghai’s metropolitan districts. It is split into training and testing: 400 images are for training and 316 for testing. The total number of pedestrians in that part is 88,488, which varies in each image from 9 to 578 with a resolution of 768 × 1024 pixels.

SmartCity: contains 50 images with 1920 × 1080 pixels of resolution [26]. Ten city scenes were used to create it, namely, an office entrance, a sidewalk, an atrium, and a commercial centre. It is comprised of both interior and outdoor scenes. The total number of pedestrians is 369, with a minimum of 1 (one) and a maximum of 14 pedestrians per image.

### 6.2. Crowded Datasets

This subsection presents an explanation of crowded datasets that have been used in the literature.

The UCF CC 50 dataset: includes various scenes such as concerts, protests, stadiums, and marathons [43]. It contains 50 images with 64 K annotated humans, the minimum and maximum number of people in the images ranges from 94 to 4543, respectively. There are 40 images in the training set and only 10 in the testing set.

WorldExpo’10: contains 1132 annotated videos from 108 surveillance cameras acquired at the Shanghai 2010 WorldExpo [59]. There are 3980 images in total in this dataset, featuring 199,923 pedestrians. Each image has between 1 (one) and 253 persons.

Shanghai part A: corresponds to the crowded part of the ShanghaiTech dataset [23], which contains 482 images collected from the internet at random. Three hundred images are used for training, and 182 images are used for testing. This subset comprises 241,677 pedestrians, where each image has a different count, ranging from 33 to 3139 persons.

The UCF-QNRF dataset: refers to a more realistic dataset since it contains diverse scenes with buildings, vegetation, sky, roads, and a diverse set of viewpoints, densities, and lighting variations [60]. It has 1535 images, and the total number of persons is 1,251,642, which ranges from 49 to 12,865 per image.

### 6.3. Evaluation Metrics

The mean absolute error (MAE) and mean squared error (MSE) are the most popular assessment metrics used in crowd counting to evaluate the performance of the proposed methods [23]. Table 4 allows one to compare the outcomes of several approaches on various datasets in regards to MAE and MSE.

MAE calculates, for each image in the dataset, the mean of the absolute differences between the actual counts and the expected counts:

(1) $$MAE=\frac{1}{N}\sum_{1}^{n}\left|z_{i}-{\hat{z}}_{i}\right|$$

MSE defines the mean of the squares of the differences between actual and estimated counts:

(2) $$MSE=\sqrt{\frac{1}{N}\sum_{1}^{n}{\left(z_{i}-{\hat{z}}_{i}\right)}^{2}}$$

In these equations, *N* is the number of test images, $z_{i}$ is the real number of persons in *i*th image, and ${\hat{z}}_{i}$ is the estimated number of persons in *i*th image.

Basically, MAE determines the accuracy of the estimations, whereas MSE determines the robustness of the forecast [23].

## 7. Results and Discussion

As an initial review of the research articles found, crowd counting is a pertinent and current topic. Regarding the review insights, the common goal in the literature is determining the number of individuals in an image, and the difference is in the model used to achieve it. Earlier studies focused on heuristic models, i.e., on detection, regression, and clustering-based methods, while recent works were more increasingly about deep learning techniques, particularly CNN based algorithms because of their robustness and performance. The results indicate that heuristic models work better on sparse datasets, while CNN models are more efficient on crowded datasets. This fact represents the reason why the decision of which model to use should consider the scenario and the number of persons in the crowd.

Nevertheless, in recent years, the researchers are more focused on deep learning based methods and have used different CNN architectures. There are three architectures that a CNN model can take: basic CNN, multi-column CNN, and single-column CNN. The challenging task in training, where perspective maps of images are not always available, is that it requires more extended training datasets due to the existence of many layers and columns.

When it comes to datasets, the researchers usually personally collected most datasets during the development of their work. Furthermore, despite differences in the number of persons, location, and whether the individuals are stationary or moving, the reviewed authors consistently use the same datasets. The differences among the used datasets can affect the results and the efficiency of the used methods. Consequently, it would be better to focus on a specific scenario and propose a model to perform better in particular situations since crowd counting tasks are implemented in different scenes for distinct goals. For example, detection-based methods are more efficient for restraint spaces, as they are faster and consume fewer resources than CNN based methods.

As an observation, researchers are following the most recent advances in deep learning by using CNN based models to solve the problem of crowd counting, especially when it comes to congested scenes. At the same time, heuristic methods such as regression and clustering, have proved their efficiency in specific scenarios. A combination of these methods in a single model could be promising.

In conclusion, this article has conducted a review of crowd counting methods from different perspectives by focusing on the architectures of the models used. It explained the different scenarios into which the datasets could be divided, and highlighted the results of the defined methods on the various scenarios addressed by the used datasets based on the top two chosen performance metrics, which allowed a benchmarking comparison.

## 8. Future Scope and Challenges

The main challenge is that crowd counting, although a topic of great interest, deals with an early stage of development. As a result, the works in literature analyze and implement various solutions, using a wide range of different models for different scenarios. Despite the considerable success of the CNN methods in terms of efficiency, the heuristic methods are still considered solutions to use in specific cases. This review highlighted the different models and the different types of datasets. The goal is to focus on diversity rather than redundancy. As a result, the comparisons in this work focused on models, their architectures, and the parameters used instead of mentioning works using the same architecture with simple modifications. However, in the upcoming work, the goal is to focus more on methods exploring the same architecture, to understand the added value that it came up with so it can inspire us in the method to implement. The idea is to combine CNN and heuristic architectures so it can be efficient for both crowded and sparse scenarios.

In the crowd counting task, most datasets contain a mix of objects such as humans, cars, and pets, which is not always the case in real-life scenarios such as in stadiums or concerts. In future work, a focus on new datasets not yet explored in crowd counting works would be essential to cover more realistic scenarios. Additionally, testing this dataset on existing models to analyze and compare the results would be crucial.

## Figures and Tables

**Table 1:** Summary of regression-based methods.

Method	Global Features	Regression Model	Dataset(s)
[44]	Segment, internal edge, texture	Gaussian	Peds1, Peds2
[45]	Segment, motion	Linear regression	PETS2009
[46]	Segment, edge, gradient	Gaussian	UCSD pedestrian, Pets 2009
[38]	Segment, edge, texture	Kernel ridge regression	UCSD, Mall
[47]	Edge	Linear regression	Internal data (2000 images, number of people per image: from 3 to 27 people)

**Table 2:** Details of the three CNN layers.

	Actions	Parameters	Input	Output
Convolutional layer	- Apply filters to extract features. - Filters are composed of learned kernels. - Apply the activation function on every value of the feature map.	- Number of kernels - Size of kernels - Activation function - Stride - Padding - Regularization type and value	- 3D cube - Previous set of feature maps	- 3D cube - One 2D map per filter
Pooling layer	- Reduce dimensionality - Extract the maximum of the average of a region. - Sliding window	- Stride - Size of a window	- 3D cube - Previous set of feature maps	- 3D cube - One 2D map per filter - Reduced spatial dimension
Fully connected layer	- Aggregate information from final feature maps - Generate final classification	- Number of nodes - Activation function	- Flattened 3D cube - Previous set of feature maps	- 3D cube - One 2D map per filter.

**Table 3:** Disadvantages and advantages of different methods that have been proposed for crowd counting.

	Disadvantages	Advantages
Detection methods	They do not perform well on crowded images as most of the target objects are not clearly visible.	They work well for detecting faces, especially for sparse datasets.
Regression methods	They always ignore spatial information.	These methods are successful in dealing with problems of occlusion and background clutter.
Clustering methods	- In the case where an individual is camouflaged, it will be ignored by the process. - Do not work for estimating crowds from individual still images.	Joint evaluation of different hypotheses is unnecessary because trajectories of trucked features are unique.
Basic CNN	Trained using perspective maps of images that are not always available.	A light network that can automatically learn the effective features for training.
Multi-column CNN	- Multi-column CNN is tough to train and takes a long time for that task. - It introduces redundant structure. - The different columns seem to behave similarly without significant differences.	Address the scale variation problem for crowd counting thanks to the use of multi-branches with different receptive field sizes.
Single-column CNN	Complex architecture for methods using encoding-decoding blocks such as TedNet.	Rather than the bloated structure of multi-column network architecture, deploys single and more profound CNNs without increasing the complexity of the network.

**Table 4:** Comparison of the performance of different methods on the used crowd counting datasets.

Methods	Year	Sparse						Crowded							
		**UCSD**		**Mall**		ShanghaiTech Part B *		**ShanghaiTech Part A**		**UCF CC 50**		**WorldExpo’10**		**UCF-QNRF**	
		**MAE**	**MSE**	**MAE**	**MSE**	**MAE**	**MSE**	**MAE**	**MSE**	**MAE**	**MSE**	**MAE**	**MSE**	**MAE**	**MSE**
MORR [38]	2012	2.29	8.08	3.15	15.7	-	-	-	-	-	-	-	-	-	-
Clustering motion cues [51]	2014	2.97	-	-	-	-	-	-	-	-	-	-	-	-	-
MCNN [23]	2016	1.07	1.35	-	-	26.4	41.3	110.2	173.2	377.6	509.1	11.6	-	277	426
CrowdNet [24]	2016	-	-	-	-	-	-	-	-	452.5	-	-	-	-	-
MSCNN [61]	2017	-	-	-	-	17.7	30.2	83.8	127.4	363.7	468.4	11.7	-	-	-
ConvLSTM-nt [62]	2017	1.73	3.52	2.53	11.2	-	-	-	-	284.5	297.1	11.9	-	-	-
CSRNet [27]	2018	1.16	1.47	-	-	10.6	16.0	68.2	115.0	266.1	397.5	8.6	-	-	-
D-ConvNet [29]	2018	-	-	-	-	18.7	26.0	73.5	112.3	288.4	404.7	9.1	-	-	-
SaCNN [26]	2018	-	-	-	-	16.2	25.8	86.8	139.2	314.9	424.8	8.5	-	-	-
CNN with pixel-wise [30]	2018	-	-	-	-	10.0	16.5	72.3	116.2	-	-	8.8	-	-	-
DecideNet [63]	2018	-	-	1.52	1.90	21.53	31.98	-	-	-	-	9.23	-	-	-
RANet [25]	2019	-	-	-	-	7.9	12.9	59.4	102.0	239.8	319.4	-	-	111	190
TedNet [28]	2019	-	-	-	-	8.2	12.8	64.2	109.1	249.4	354.5	8.0	-	113	188
PaCNN [64]	2019	0.89	1.18			8.9	13.5	66.3	106.4	267.9	357.8	7.8			
SAAN [65]	2019	-	-	1.28	1.68	-	-	-	-	-	-	-	-	-	-
PGCNet [66]	2019	-	-	-	-	8.8	13.7	57.0	86.0	-	-	8.1	-	-	-
ADSCNet [67]	2020	-	-	-	-	6.4	11.3	55.4	97.7	-	-	-	-	71.3	132.5
SASNet [68]	2021	-	-	-	-	6.35	9.9	53.59	88.38	161.4	234.46	5.71	-	85.2	147.3

* ShanghaiTech Part B is a sparse dataset that is why it is mentioned before Part A in the table.

## Data Availability

Not applicable.
